# The net return from animal activity in agro-ecosystems: trading off benefits from ecosystem services against costs from crop damage

**DOI:** 10.12688/f1000research.2-239.v2

**Published:** 2014-04-30

**Authors:** Gary W Luck

**Affiliations:** 1 Institute for Land, Water and Society, Charles Sturt University, Albury, 2640, Australia

## Abstract

Animals provide benefits to agriculture through the provision of ecosystem services, but also inflict costs such as damaging crops. These benefits and costs are mostly examined independently, rather than comparing the trade-offs of animal activity in the same system and quantifying the net return from beneficial minus detrimental activities. Here, I examine the net return associated with the activity of seed-eating birds in almond orchards by quantifying the economic costs and benefits of bird consumption of almonds. Pre-harvest, the consumption of harvestable almonds by birds cost growers AUD$57.50 ha ^-1^ when averaged across the entire plantation. Post-harvest, the same bird species provide an ecosystem service by removing mummified nuts from trees that growers otherwise need to remove to reduce threats from fungal infection or insect pest infestations. The value of this ecosystem service ranged from AUD$82.50 ha ^-1^–$332.50 ha ^-1^ based on the replacement costs of mechanical or manual removal of mummified nuts, respectively. Hence, bird consumption of almonds yielded a positive net return of AUD$25–$275 ha ^-1^ averaged across the entire plantation. However, bird activity varied spatially resulting in positive net returns occurring primarily at the edges of crops where activity was higher, compared to negative net returns in crop interiors. Moreover, partial mummy nut removal by birds meant that bird activity may only reduce costs to growers rather than replace these costs completely. Similar cost-benefit trade-offs exist across nature, and quantifying net returns can better inform land management decisions such as when to control pests or promote ecosystem service provision.

## Introduction

Animals provide benefits to humans through ecosystem services including the provision of food and fibre, crop pollination, biological control, waste disposal, nutrient cycling and seed dispersal ^1– 5^. Animal behaviour also inflicts costs on humanity, particularly through damage to food crops grown for human consumption ^6– 9^. The monetary value of the benefits and costs of animal activity can be substantial. For example, Losey and Vaughan ^10^ estimated that the annual value of wild pollinators to agriculture in the United States (US) was approximately US$3 billion, while natural pest control services were worth about US$13.6 billion annually. Conversely, crop damage caused by the European starling ( *Sturnus vulgaris*) in the US costs around US$800 million each year ^11^, while bird damage to horticultural production in Australia is estimated at AUD$300 million annually ^12^.

Despite the obvious cost-benefit trade-offs of animal activity, studies on the ecosystem services provided by animals and on the damage they cause have evolved largely independently ^13^. Yet, to more accurately reflect the outcomes of animal activity for society, it is imperative to quantify and compare the costs and benefits of these activities in the same system. Cost-benefit trade-offs are most acute in agricultural systems ^14^, which profit from a range of animal-based ecosystem services, but suffer also from substantial negative impacts from animal activity.

The net return associated with animal activity in a given system can be derived by subtracting the costs of this activity (damage inflicted) from the benefits (ecosystem services provided). Benefits and costs may be quantified in monetary terms or some other appropriate metric (e.g. net effect on crop yield). A net return can be either positive or negative depending on the difference between the value of the ecosystem service(s) and the value of the negative impact(s). This approach is fundamentally different to other cost-benefit trade-offs presented in the literature, such as trading off the cost of supporting ecosystem service providers (e.g. by planting or protecting their habitat) against the value of the services provided ^15^, or comparing the cost of pest control strategies with the amount of damage inflicted by pests ^16^. Conceptually, it is most similar to circumstances where particular animals inflict damage (e.g. insect pests), other animals help control these pests (e.g. insectivorous birds), and researchers compare crop yield with and without the ecosystem service providers (e.g. Mols and Visser ^2^, and Kellerman ^3^). Focussing on net return, however, shifts the emphasis to quantifying both the costs and the benefits of the activity of particular animals, subtracting one from the other, and includes cost-benefit trade-offs stemming from the activity of the *same* species or the same group of species.

**Figure 1.**
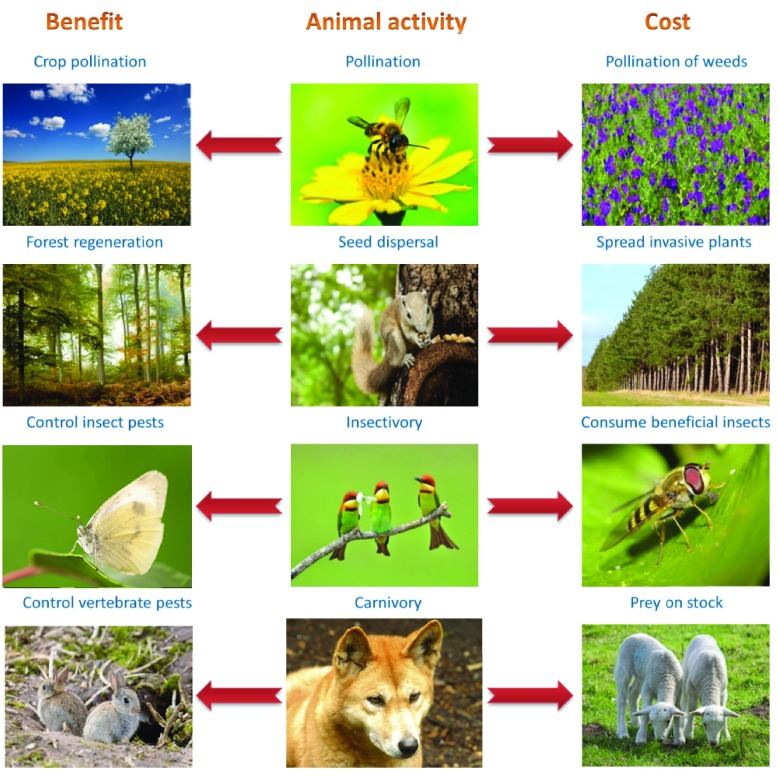
The same animal activity can confer benefits and costs on society. These cost-benefit trade-offs are common across nature. A positive net return from animal activity occurs when benefits outweigh costs, with the converse resulting in a negative net return. Photo credits: FreeDigitalPhotos.net (top left: prozac1, top middle: sweetcrisis, top right: John White, 2nd row left: dan, 2 ^nd^ row middle: artemisphoto, 2 ^nd^ row right: xedos4, 3 ^rd^ row middle: thawats, 3 ^rd^ row right: Paul Brentnall; bottom row right: Dr Joseph Valks). Remaining photos are from thinstockphotos.co.uk.

Quantifying net returns is applicable to many situations within agriculture and more broadly ( Figure 1). For example, pollinating insects contribute substantially to the pollination of food crops ^17^, but the same insects may also pollinate agricultural weeds ^18^. In a given location, the net return of pollinator activity could be quantified by comparing crop yield from pollination vs. reductions in yield caused by competition from insect-pollinated weeds. Similarly, insectivorous animals may help control insect pests in crops ^2^, but could also consume desirable insect species (e.g. pollinators) ^19^. An analysis of insectivore activity, diet composition, and changes in crop yield with variation in animal activity and abundance could be conducted to quantify this cost-benefit trade-off.

**Figure 2.**
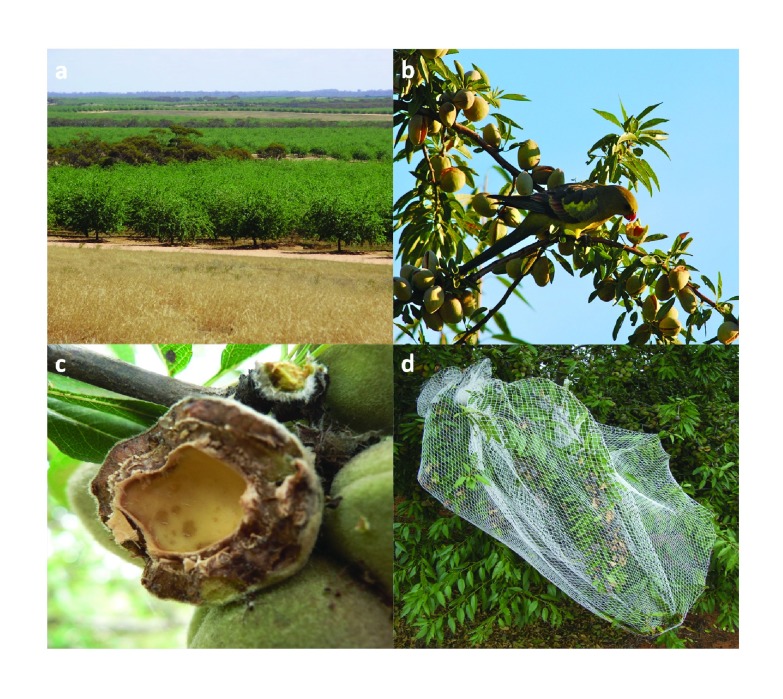
( **a**) Almond orchards in north-west Victoria, Australia have expanded 10-fold in 10 years, now covering more than 20,000 ha. ( **b**) A regent parrot ( *Polytelis anthopeplus*) feeding on almonds. Regent parrots are one of 11 parrot and cockatoo species that have been recorded eating almonds. ( **c**) Typical parrot damage to almonds. ( **d**) A netted branch on an almond tree. Photo credits: Hugh McGregor and Shannon Triplett.

In this study, I present the first ever field test of this net return approach by quantifying the economic costs and benefits of bird activity in almond orchards in southern Australia ( Figure 2). Almonds are one of Australia’s fastest growing horticulture sectors. The area of almond plantations increased 5-fold between 2001 and 2011, from 5,900 ha to over 30,000 ha, and annual production is projected to reach 88,000 tonnes in 2016, up from 37,000 tonnes in 2011 ( http://www.australianalmonds.com.au/industry). Australia will soon be the world’s second largest almond producer behind California, USA.

The expansion of the almond industry raises substantial production and conservation management challenges. In Australia, almond crops attract a number of native bird species, especially parrots, which eat almonds during the growing season and reduce crop yield ^20^. However, the same bird species provide an ecosystem service to growers by eating residual nuts left on trees after the main crop has been harvested. These so-called ‘mummified nuts’ (mummy nuts) are susceptible to fungal infection, which may threaten future crop yields. Moreover, recent evidence shows that mummified nuts are used intensively by the carob moth ( *Ectomyelois ceratoniae*) for food and breeding ( http://australianalmonds.com.au/industry/conference_2012/proceedings). The carob moth is a pest of global significance, impacting the production of numerous crops worldwide including dates, figs, pistachio, citrus and pomegranate ^21– 23^. It is a major emerging threat to the Australian almond industry, as it feeds on almond kernels rendering them unsuitable for human consumption. Mechanical or manual removal of mummy nuts post harvest is one approach to controlling moth outbreaks and fungal infections. However, birds are already providing this service to Australian growers – the question addressed in my study is whether the monetary value of this service outweighs the costs of bird damage to almonds, resulting in a positive net return from bird activity.

## Methods

My study was conducted in almond plantations in north-west Victoria, Australia, centred on 34°45′00S 142°42′52E ( Figure 2a). This was a two-phrase experiment based on the exclusion of birds from almond trees. The first phase quantified damage to ripening nuts – the ‘cost’ component of the cost-benefit trade-off. The second phase quantified the removal of mummy nuts by birds post-harvest – the ‘benefit’ component. Experiments were conducted in two almond blocks (~ 17 ha each) in a single plantation of even-aged trees (~ 8 years old) of the same almond variety (nonpareil). At least 13 bird species have been recorded feeding on almonds by local growers ( Table 1), including the threatened regent parrot ( *Polytelis anthopeplus*) ( Figure 2b) ^20^.

**Table 1.** Bird species recorded feeding on almonds in plantations in north-west Victoria, Australia.

Common name	Scientific name
Australian raven	*Corvus coronoides*
Australian ringneck	*Barnardius zonarius*
Blue bonnet	*Northiella haematogaster*
Eastern rosella	*Playtcercus eximius*
Galah	*Eolophus roseicapillus*
Little corella	*Cacatua sanguinea*
Little raven	*Corvus mellori*
Long-billed corella	*Cacatua tenuirostris*
Mulga parrot	*Psephotus varius*
Red-rumped parrot	*Psephotus haematonotus*
Regent parrot	*Polytelis anthopeplus*
Sulphur-crested cockatoo	*Cacatua galerita*
Yellow rosella	*Platycercus elegans flaveolus*

### Costs

To quantify bird damage to ripening nuts, trees were netted using 15 mm diamond mesh bird exclusion netting during the growing season of 2010/11. Nets were placed in October following natural early abortion of nuts by trees, and remained on trees until harvest (~ March). A total of 120 trees were included in the experiment with 60 netted trees (treatment) and 60 open trees (control). Thirty netted trees and 30 open trees were located at the edge of almond blocks (the exterior two rows) and 30 netted trees and 30 open trees were located in the interior of almond blocks (the centre point furthest from the edge – approximately 300 m). An edge/interior contrast was included because bird activity tends to be highest close to block edges ^20^. Control and treatment trees were assigned systematically to maximize interspersion ^31^, and I maintained a minimum distance of four trees between each control and treatment in the same edge or interior row to avoid adjacency effects and spatial autocorrelation in damage impacts (e.g. netted trees impacting outcomes on non-netted trees). The purpose of netting trees was to record levels of nut loss attributable to factors other than birds (e.g. storm damage, tree health and natural abortion). This enabled me to partition out nut loss attributed to either birds or non-bird factors.

I netted a single, randomly chosen lateral branch on each treatment tree rather than attempt to net the whole tree ( Figure 2d; trees were divided into three height sectors (lower, middle and upper) and four quadrants based on cardinal N, S, E and W, and a height sector and quadrant was chosen at random to select target branches). A lateral branch on each control tree was also chosen at random and these were completely open to birds. At the beginning of the experiment, almonds on each lateral branch were counted (98 nuts branch ^-1^, ± 1 SEM 3.5, *n* = 120). At the end of the experiment immediately prior to harvest, nuts were assigned to one of the following three categories: intact on branch; damage/loss attributed to birds; damage/loss attributed to non-bird factors.

Netting whole trees was problematic and could have compromised the integrity of the experiment in several ways. Netting the entire tree would have disrupted regular orchard maintenance such as selective pruning and the spraying of herbicides to control weed growth. Hence, netted trees would have differed from non-netted trees in more than just the presence of a net. Second, strong wind is locally common and can be particularly problematic at orchard edges, which have little protection from the elements. Given the structure of almond trees, and the closeness of plantings, there was a real threat of nets ripping during strong winds if placed over the entire tree. Third, the larger the area netted, the more likely that birds will find gaps in netting. It is extremely difficult to ensure that birds are completely excluded from an entire tree even when careful attention is paid to closing all visible gaps in the net. Owing to these concerns, I decided that netting a single branch was a more acceptable method to employ. I do not believe that this approach undermines the general conclusions of the study.

Damage to almonds by birds is readily identifiable through bite patterns ^20^. While the hulls of nuts often remain on the tree after bird damage (see Figure 2c), some nuts may be completely removed by birds. In this case, it is difficult to attribute nut loss to birds or non-bird factors that may result in complete nut loss (e.g. storm damage). Nevertheless, I used data from netted trees to calculate mean percent nut loss from non-bird factors and adjusted values for open trees. Mean nut loss from netted trees (i.e. netted branches) was 5.9% (± SEM 0.82%, n = 60), so if, for example, 50 nuts were completely lost from an open tree [branch], I considered that three nuts (5.9%) were lost due to non-bird factors and the remainder due to birds. I did not expect netting to affect non-bird related nut loss.

The monetary cost of bird activity was measured as reduced yield in the almond crop (damage or loss), converted to an AUD$ value based on the wholesale almond price for 2012 of $5.05 kg ^-1^. The average weight of a raw, shelled almond is 1.3 grams (± SEM 0.005 grams, *n* = 500), so at a price of $5.05 kg ^-1^ a single almond is worth approximately $0.007. Hence, a 10% yield loss from an open lateral branch with a starting crop of 100 almonds equates to $0.07 (10 nuts lost or damaged). I was able to extrapolate yield loss values from lateral branches to values per tree based on the mean number of nuts per tree (1268, ± SEM 43.6, *n* = 200), which was estimated as part of a related study on bird damage to almonds ^20^. Based on the value of $0.007 nut ^-1^, a 10% yield loss for an entire tree equates to $0.83 (± SEM $0.03). Per tree values were converted to per ha values using the recommended almond planting density of 250 trees ha ^-1^ ( http://www.dpi.vic.gov.au/agriculture/horticulture/fruit-nuts/nuts/almonds).

### Benefits

Post-harvest, the same experimental design was employed to quantify the removal of mummy nuts left on trees (i.e. 60 netted and 60 control trees split evenly between the edge and interior of two almond blocks). Different trees and different lateral branches were used in this experiment, as I had to target trees and branches that retained mummy nuts. Nets remained on the trees for approximately 3 months, from March to June. Nuts were counted at the beginning and end of the experiment and assigned to one of the three categories listed above.

To quantify the monetary value of bird removal of mummy nuts, I used the replacement cost method. This method determines the economic value of an ecosystem service by calculating the cost of replacing that service via human-derived means. It has been used widely to estimate the replacement costs of ecosystem services provided by particular ecosystems (e.g. forests or wetlands; see for example Spangenberg and Settele ^32^, Turner *et al.* ^33^, and Zhang *et al.* ^34^), but also for services provided by particular species ^1, 35^. While the use of this method under certain circumstances has been criticized (e.g. Winfree *et al.* ^36^), it was the most appropriate approach in my study because mechanical or manual removal of mummy nuts by growers is the currently employed and least costly alternative to bird removal of mummy nuts.

Mummy nuts can be removed by shaking the almond tree using a large mechanical shaker (akin to a large tractor) or hand-poling, which involves a labourer knocking nuts from the tree using an elongated pole (manual labour). Mechanical removal, including sweeping and shredding of nuts, takes approximately 20 seconds per tree. The cost of this was converted to an hourly rate based on the hourly wage of a skilled mechanical-shaker driver plus a retail hire rate for large farm machinery ($38 hr ^-1^). In Australia, the full-time minimum wage for adults in 2012 was $15.96 hr ^-1^. I assumed skilled operators of large machines would attract a higher hourly rate than the minimum wage, and based my initial calculations on a nominal value of $20 hr ^-1^ (see Results for cost-benefit trade-offs across different wage rates).

Hand-poling is more labour and time intensive than mechanical shaking, but requires less training. It can be used instead of or in addition to mechanical shaking if the latter method is unable to dislodge the majority of mummy nuts. The main cost of hand-poling is associated with the time taken to reduce the number of mummy nuts on an almond tree to an acceptable level (see Discussion). Assuming a minimum wage for hand-polers of $15.96 hr ^-1^, 1 minute of hand-poling per tree costs $0.27 ($67.50 ha ^-1^).

I determined if there was a block or row location (edge/interior) effect on nut loss attributed to birds or non-bird factors pre- and post-harvest using a generalized linear model with a binomial response. In this case, the binomial outcome was either nut damaged/lost vs. nut intact. That is, I compared nut outcome among almond blocks and edge and interior rows pre-harvest and post-harvest for open trees where the outcome could be either nut damaged/lost to bird activity or nut remained intact. I conducted the same analysis for netted trees pre- and post-harvest where nut outcome was affected by non-bird factors (e.g. wind). Row location was nested within block for these analyses and both were treated as random factors and p ≤ 0.05 was considered statistically significant. Analyses were conducted using SPSS Version 21.0 ^37^.

## Results

**Figure 3.**
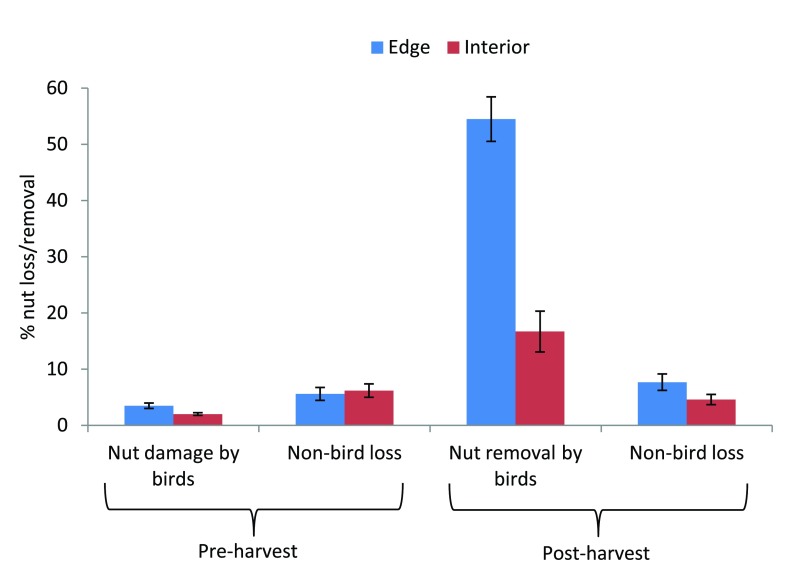
Almond damage and mummy nut removal. The percentage of almonds damaged or removed by birds, or lost through non-bird factors, at the edge and interior of almond blocks pre- and post-harvest. Error bars are ± 1 SEM; *n* = 120 both pre and post-harvest, split evenly among treatments.

Bird damage to almonds pre-harvest was generally low, averaging 2.8% (± 1 SEM 0.28%) of the crop. Damage was higher at the edge of almond blocks compared to the interior ( *χ*_1_^2^ = 16.18, *P* < 0.001; Figure 3), but there was no difference between almond blocks. Nut loss attributed to non-bird factors (e.g. storms, tree health, natural abortion) was more than two times greater than bird damage (5.9% ± SEM 0.82%) and differed between almond blocks ( *χ*_1_^2^ = 38.04, *P* < 0.001, higher in block 1), but not between row location ( Figure 3). Post-harvest, mummy nut removal by birds was more than 10 times greater, on average, than bird damage pre-harvest (35.6% ± SEM 3.6%). Nut removal was much higher at block edges compared to the interior ( *χ*_1_^2^ = 224.5, *P* < 0.001; Figure 3) and also differed between almond blocks ( *χ*_1_^2^ = 5.89, *P* = 0.02, higher in block 1). Nut loss via non-bird factors post-harvest was 6.1% (± SEM 0.9%) and was higher at block edges ( *χ*_1_^2^ = 3.9, *P* = 0.05).

Based on an almond wholesale value of $5.05 kg ^-1^, average bird damage pre-harvest (2.8%), across the entire plantation, cost growers $0.23 tree ^-1^ or $57.50 ha ^-1^ (± SEM $5; all amounts in AUD). Nut loss from non-bird factors cost more than double the loss attributed to birds ($122.50 ha ^-1^, ± SEM $17.50). Yield loss from bird or non-bird factors represents 0.8% and 1.6%, respectively, of crop value ha ^-1^ based on a ‘good’ yield of 1.5t ha ^-1^ of almonds at current wholesale value ( http://www.dpi.vic.gov.au/agriculture/horticulture/fruit-nuts/nuts/almonds).

**Figure 4.**
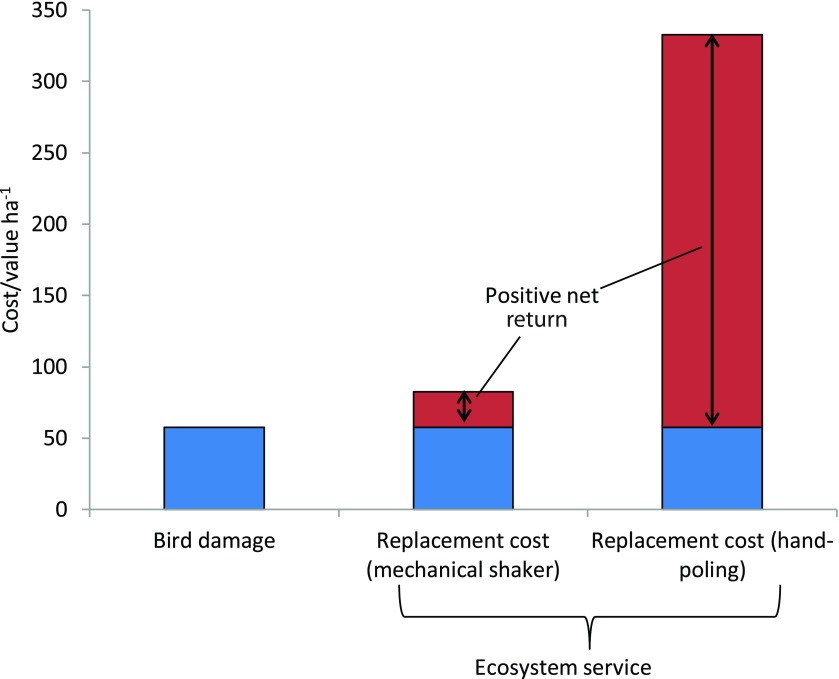
The cost of bird damage to almonds pre-harvest compared to the value of the ecosystem service post-harvest (removal of mummy nuts). Ecosystem service value is calculated using the replacement cost method based on the removal of mummy nuts via mechanical shaking or hand-poling. A positive net return (red) occurs when the ecosystem service value is greater than the cost of bird damage (blue).

Using a mechanical tree shaker, it costs growers $82.50 ha ^-1^ to remove mummy nuts (incorporating salary ($20 hr ^-1^) and machinery costs). A more generous salary of $30 hr ^-1^ raises costs to $95 ha ^-1^. Manual removal of mummy nuts using hand-poling is more expensive. Assuming only a 5-minute duration at each tree and a minimum wage of $15.96 hr ^-1^, hand-poling costs $332.50 ha ^-1^. If bird consumption reduces the number of mummy nuts to an acceptable level, negating the need for growers to remove nuts, the value of this ecosystem service outweighs the costs of crop damage by at least $25–$275 ha ^-1^ (replacement costs of mechanical shaking and hand-poling, respectively). Hence, bird activity yields a positive net return ( Figure 4).

**Figure 5.**
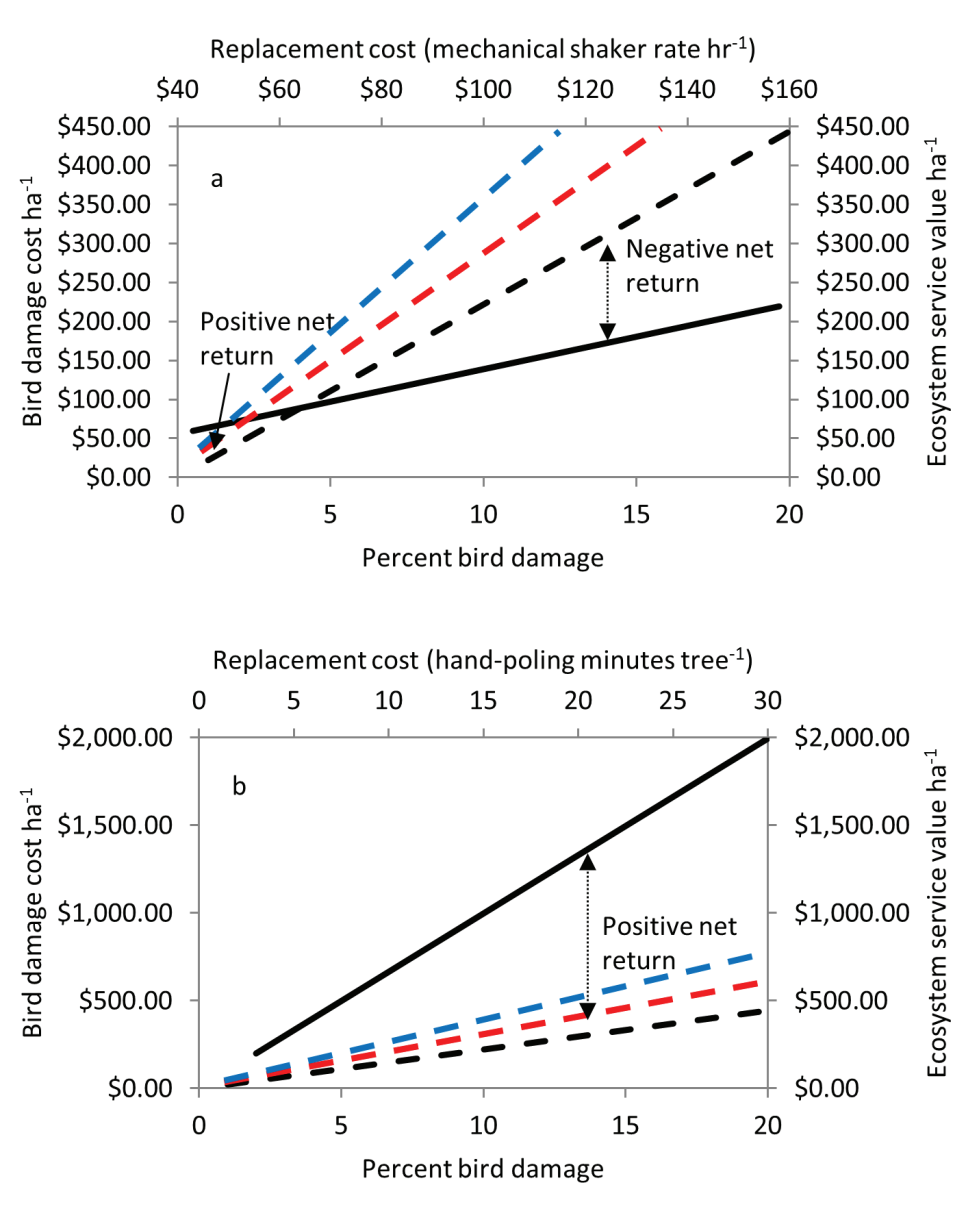
( **a**) Comparing the trade-off in the cost of bird damage (dashed line) vs. the value of the ecosystem service of mummy nut removal (solid line) with variation in damage rates and replacement cost estimates based on mechanical shaking. Higher rates of bird damage will generally result in negative net returns for growers even at relatively high replacement costs (ecosystem service value). ( **b**) The same trade-off using replacement cost estimates from hand-poling (solid line) suggests that even high rates of bird damage (dashed line) will not outweigh ecosystem service value, consistently yielding a positive net return. The red dashed line shows relationships based on the expected 2013 market price for almonds ($6.38 kg ^-1^) and the blue dashed line is based on a hypothetical market price ($8.04 kg ^-1^) assuming growth in market value from 2012–13 is repeated for 2013–14.

Whether a positive or negative net return occurs depends on the level of bird damage to crops, the market value of almonds, and the value of the ecosystem service. I calculate that a ‘break even’ point, where damage costs and ecosystem service value are about equal, occurs when bird damage is 4% and the value of mummy nut removal is $87.50 ha ^-1^ based on the replacement cost of mechanical shaking ($63 hr ^-1^; Figure 5a). Damage costs increase steadily beyond this point and always exceed ecosystem service value even when the replacement cost is high, yielding negative net returns for growers. However, if the value of the ecosystem service is based on the replacement cost of hand-poling mummy nuts, then the benefit outweighs the cost of bird damage (a positive net return) even if only 10 minutes or less is spent hand-poling each tree and bird damage rates equal 20% ( Figure 5b). These relationships vary when considering different market prices for almonds.

These averaged results are complicated by two factors. First, bird damage and mummy nut removal varied spatially – both being higher at the edge compared to the interior of almond blocks. Pre-harvest, bird damage at the edge of blocks averaged 3.5% equating to a cost to growers of $72.75 ha ^-1^, while lower damage in the interior (2%) equals a cost of $41.50 ha ^-1^. Second, the averaged values (across the entire plantation) assume that birds will remove most mummy nuts within a given time frame, negating the need entirely for mechanical or manual removal by growers. For the 3-month experiment, birds removed 36% of mummy nuts, on average, per tree. If ecosystem service value is calculated using a reduced cost estimate (rather than using replacement costs), then growers would save $30 ha ^-1^ if only having to remove 64% of mummy nuts by mechanical shaking (i.e. $82.50 ha ^-1^ to remove 100% of nuts minus $52.50 ha ^-1^ to remove 64% of nuts), and $120 ha ^-1^ by hand-poling.

For mechanical shaking, the ecosystem service value of partial mummy nut removal does not outweigh the cost of bird damage, but it does for hand-poling. Assuming a linear rate of mummy nut removal by birds over time, if growers are able to wait until the next almond flowering (approximately 5 months based on harvesting in February/March and flowering in July/August), then birds would have removed 60% of mummy nuts. In this instance, growers would save $52.50 ha ^-1^ for mechanical shaking, making the ecosystem service value only marginally less than the cost of bird damage.

However, at the edge of almond blocks, mummy nut removal over 3 months equalled 55%, equating to over 90% of nuts removed within 5 months assuming a linear rate of removal. In this instance, mechanical or manual removal may be completely unnecessary and even with the higher rate of bird damage there is still a positive net return from bird activity of at least $9.75 ha ^-1^. This suggests that growers need to consider possible spatial variation in cost-benefit trade-offs. It is also important to note that my analysis assumes that a single visit per tree by the mechanical shaker is enough to remove the majority of mummy nuts. This is unlikely (see Discussion), resulting in a conservative estimate of ecosystem service replacement costs in this instance.

Pre-harvest nut damage and post-harvest mummy nut removal by birds in an Australian almond plantation. Pre-harvest nut damage. Contains data on the number of almonds on each lateral branch at the beginning of the experiment (‘Total almonds’), the number eaten by birds (‘Eaten by birds’), and the percentage of total nuts damaged (‘percent damaged’) for trees exposed to birds (‘Open trees’) at two almond blocks (‘Almond block; 1, 2’) in either edge or interior block rows (‘Row location; edge, interior’). Data are also provided for trees excluding birds (‘Netted trees’) showing the number of almonds on each lateral branch at the beginning of the experiment (‘Total almonds’), the number lost to non-bird factors (e.g. storms) (‘Non-bird nut loss’), and the percentage of total nuts lost (‘percent loss’) at two almond blocks in either edge or interior block rows.
Post-harvest mummy nut removal. Contains data on the number of mummy nuts on each lateral branch at the beginning of the experiment (‘Total mummy nuts’), the number eaten by birds (‘Eaten by birds’), and the percentage of total nuts eaten (‘percent eaten’) for trees exposed to birds (‘Open trees’) at two almond blocks (‘Almond block; 1, 2’) in either edge or interior block rows (‘Row location; edge, interior’). Data are also provided for trees excluding birds (‘Netted trees’) showing the number of mummy nuts on each lateral branch at the beginning of the experiment (‘Total mummy nuts’), the number lost to non-bird factors (‘Non-bird nut loss’), and the percentage of total nuts lost (‘percent loss’) at two almond blocks in either edge or interior block rows.
Data shows the relationship between % bird damage to the almond crop ('percent bird damage') and the cost to growers ('AUD cost/ha') based on a wholesale almond price of $5.05 kg-1 (dashed line in Figure 5a in main article). The relationship between the cost of replacing the ecosystem service based on varying the salary rate for mechanical shaker drivers - 'Replacement cost (salary rate/hr)' and the ecosystem-service value (bold line in Figure 5a in main article). Also shown is the relationship between the cost of replacing the ecosystem service based on varying the time spent hand-poling a tree where salary rates are held constant at $15.96 hr-1 - 'Replacement cost (minutes /tree)' and the ecosystem-service value (bold line in Figure 5b in main article). All cost values are in US dollars.

## Discussion

On average, bird activity yielded a positive net return to almond growers, even with relatively low ecosystem service replacement costs. The replacement cost estimate of mechanical shaking is conservative because I assumed shaking will remove most mummy nuts in a single visit, but this may not be the case; mummy nuts occur because mechanical shaking during harvesting does not dislodge every almond from a tree ^24^. Moreover, mechanical shaking may impact the following season’s yield if conducted just prior to bud development ^25^. Therefore, it is likely that a combination of mechanical shaking and hand-poling, or hand-poling alone, is required to guarantee that most mummy nuts are removed. Owing to the higher costs of hand-poling, the ecosystem service value of mummy nut removal by birds always yielded a strong positive net return, even when damage rates pre-harvest were high, and even when ecosystem service value was calculated as the amount saved by growers if only having to remove ~ 60% of mummy nuts.

Nevertheless, it is important to recognise the spatial variation that occurred in bird activity and subsequent cost and benefit outcomes, and the fact that birds did not remove all mummy nuts. The value of the ecosystem service provided by birds relates to what is an ‘acceptable’ level of mummy-nut load remaining on trees post-harvest. In California (the world’s major almond growing region), it is generally considered that approximately two nuts per tree is an acceptable quantity of mummy nuts to reduce adverse impacts ( http://www.ipm.ucdavis.edu/PMG/C003/m003dcmummynut.html). I am unaware of any similar guidelines for Australia, possibly because threats associated with mummy-nut retention (e.g. carob moth infestations) are relatively new, but it is important to recognise that bird activity is unlikely to remove all mummy nuts from trees before onset of the next crop. Therefore, bird activity may at best reduce costs for growers rather than replace these costs completely, and this reduction is likely to be highest at crop edges. Nevertheless, if the acceptable quantity of mummy nuts that can be retained per tree is substantially higher than two, then the cost of mummy nut removal by growers is reduced even further.

The fact that birds did not remove all mummy nuts prior to the next almond flowering highlights the important point that ecosystem-service provision in this case does not replace completely the need for human input to achieve acceptable outcomes. A combination of human action and ecosystem-service provision is required, and I suggest this is likely to be the case in many different systems. It is important to determine, therefore, how the proportional contribution required from human action or other organisms changes with different environmental, social and economic conditions. For example, in my study, because bird activity was highest at crop edges, the proportional contribution by farm managers to mummy nut control may be minor in this location, but increase further from the edge.

Information about the magnitude of positive or negative net returns can be used by agriculturalists to better inform crop management decisions, such as when and how to control pests. The trade-off between the monetary costs of pest damage vs. how much to invest in pest control is a common problem in agriculture ^16^. Yet, such analyses would benefit immensely if they considered also the value of any services provided by the ‘pest’ species or other species in the system. If a positive net return from animal activity is recorded in a given agro-ecosystem, pest control activities may be unnecessary or even detrimental. Conversely, if a negative net return is recorded, the magnitude of the negative return could be used to guide pest control spending.

In almond plantations, birds are currently considered pests and are subject to control strategies such as shooting to scare. In my study, shooters were employed for an average of 120 days per season over the growing seasons of 2009/10 and 2010/11. Based on an 8-hr day and minimum wage ($15.96 hr ^-1^), the cost of employing shooters is at least $15,322 season ^-1^. Over a 15,500 ha plantation (the entire plantation estate for my study) this equates to about $1 ha ^-1^, and appears to be a good investment if compared only to the cost of bird damage. For example, if shooting reduces bird damage by only 1% it would save growers $22.19 ha ^-1^. Yet, in the context of an overall positive net return from bird activity, the investment in shooting may be completely unnecessary.

It could be argued that a positive net return was recorded in my study only because of current pest control strategies. However, our previous work ^20^ and research more generally ^26, 27^ shows that shooting is largely ineffective at controlling bird behavior. Moreover, if season-long control strategies are effective in reducing bird use of almonds, they may disrupt the ecosystem service being provided post-harvest.

Agriculturalists may also invest in improving service provision in addition to or instead of controlling pests. In my study system, birds appear to use almonds when alternative food resources are scarce ^20^. Plantings of decoy crops (e.g. low value crops or stands of native forage plants) ^28, 29^ that provide food during the almond growing season could be used to reduce bird impacts. In my study area, native plants from genera such as *Atriplex, Eremophila, Dodonaea* and the family Amaranthaceae (e.g. chenopods) should be considered for decoy crops. Moreover, strategic planting coupled with plant phenology could ensure that food resources from decoy crops were near exhausted post almond harvest, resulting in birds moving into orchards to feed on mummy nuts. Parrot density in almonds is already higher late in the season, as more almonds have split hulls and kernels are easier to access ^20^. This is why removal rates for mummy nuts were much higher than damage rates pre-harvest in my study ( Figure 3). Hence, a single management action – planting decoy crops with appropriate phenology – could simultaneously reduce bird damage to almond crops while maintaining and possibly improving the service of mummy nut removal.

Agricultural land-use management strategies must consider the cost-benefit trade-offs occurring across the spectrum of animal activities and their impacts. This approach is fundamentally different to current research that calculates only the benefit of an ecosystem service provided by animals, even when that service is the control of another animal pest. In these case studies, the benefit is usually measured as the difference in crop yield with and without the provision of the service (e.g. Mols and Visser ^2^, Kellerman ^3^, Karp *et al.* ^38^). While difference in yield could be considered the net outcome of the activities of the animal pests and their control agents, critical information is usually not considered such as the cost of any damage inflicted on crops by those species providing the ecosystem service or benefits provided by the ‘pest’ species. That is, it is important to acknowledge that an ecosystem-service provider may be a pest in a different context, and vice-versa for recognised pest species. This information is vital to guiding more strategic land management decisions regarding monetary investments in the control of pests or supporting the provision of ecosystem services.

It is also important to note that the sign and the magnitude of the net return of animal activity will vary with fluctuations in the market prices for crops and the costs of replacing the ecosystem service (e.g. the running costs of machinery and the salary costs of orchard workers). An analysis of how cost-benefit trade-offs change with varying monetary values is provided in Figure 5, including trend lines based on different market prices of almonds. Researchers that adopt the approach of calculating a net return from animal activity should use the most recent and relevant monetary values for their specific situation.

The specific results of the case study I present here may not be transferable across different contexts. Nevertheless, the need to calculate a net return from animal activity is broadly relevant because there are few situations in nature whereby the activity of animals in a given location is entirely beneficial or entirely detrimental – especially when considering a broad range of taxa.

I’ve focussed on a small suite of species and a single activity to clearly illustrate the net return from animal activity. Analyses could be extended to encompass more species and more activities, dependent on available ecological knowledge. For example, I have recorded 35 insectivorous bird species using almond orchards in my study area that may contribute to controlling carob moth. Almond flowers require cross pollination to set seed, and in north-west Victoria, over 100,000 European honeybee ( *Apis mellifera*) hives are trucked into almond plantations each year, costing growers more than $7 million annually. Yet, almost nothing is known of the potential contribution that native pollinators could make to almond pollination in this region ^30^. A more complete analysis could consider the costs and benefits, and ultimately net return, of the activities of a range of species.
