# Supplementary material for: The net return from animal activity in agro-ecosystems: trading off benefits from ecosystem services against costs from crop damage
Source: F1000Res. 2014 Apr 30;2:239. Originally published 2013 Nov 12. [Version 2] doi: 10.12688/f1000research.2-239.v2 (PMC4176426; doi:10.12688/f1000research.2-239.v2)
Supplement: Peer review history

# Peer review: Referee response for version 2

**DOI:** 10.5256/f1000research.4324.r5204

**Published:** 2014-06-20

**Authors:** Stijn Reinhard

**Affiliations:** 1 Agriculture Economics and Rural Policy, Wageningen University and Research Center, Wageningen, Netherlands

I agree with most of the responses from Gary Luck.

- I prefer to persist in my sixth remark on the elaboration of the Generalized Linear Model. The explicit specification of this model and the estimation results are missing. In his response the author describes the method and its results in words. I believe the paper should contain the quantitative estimation results too.
- In my ninth remark I mentioned the partial analysis. The author has responded by providing an array of other relevant ecosystem services for almond orchards. Indeed, a more comprehensive study will shed light on the value of other ecosystem services for almond orchards. It will also be interesting to see whether these parrots provide other ecosystem services than those for almond orchards.

I have read this submission. I believe that I have an appropriate level of expertise to confirm that it is of an acceptable scientific standard.

# Peer review: Referee response for version 1

**DOI:** 10.5256/f1000research.2837.r4503

**Published:** 2014-04-16

**Authors:** Stijn Reinhard

**Affiliations:** 1 Agriculture Economics and Rural Policy, Wageningen University and Research Center, Wageningen, Netherlands

The research seems to be performed well, but some remarks can be made with regard to the description and application of the research results.

1. The objective of the paper is to quantify the costs and benefits of animal activity for society.  Animal activity is related to ecosystem services. In this paper one ecosystem service is analysed: birds eating almonds in almond orchards in southern Australia. When birds eat pre-harvest almonds it is considered a cost and post-harvest consumption of almonds is regarded a benefit of bird activity. The benefits are associated with the prevention of fungal infection. Hence, the title is somewhat misleading. It should be more precisely: The net return from animal activity in agro-ecosystem services: trading off costs and benefits from ecosystem services.
2. For an economic analysis, the context of the research objective should be clear. Are birds considered a pest by farmers (or the society) and are they looking for measures to reduce the nuisance. In that case the optimal measure to reduce the costs of bird eating almonds could be computed using a cost benefit analysis, taking into account various measures.
3. A costs benefits analysis is usually estimated for the ex-ante evaluation of a project. The project is not clear in this paper. Costs are computed based on an analysis of branches of almond trees that are netted to prevent birds eating almonds before harvest. But although it is not explicitly stated in the paper, netting entire trees does not seem to be an attractive measure. Shooting to scare is mentioned as a measure, but this measure is not analysed (as a difference between shooting and no-shooting). It seems that shooting was practiced during the analysis, the impact of shooting on the results is not analysed explicitly.
4. The benefits of almond removal by birds are calculated based on various assumptions on nut removal by the farmer (mechanically or by hand) and on the (labour) costs associated with this nut removal. Using actual (bookkeeping) information of the orchard (or similar orchards) would lead to a more robust analysis.
5. The real benefit is the prevention of fungal infection. The author is not aware of Australian guidelines on the acceptable quantity of mummy nuts to prevent infection. His assumption that 2 nuts per tree is acceptable will affect the confidence interval of the results.
6. The ‘generalized linear model with a binominal response’ used (page 5) needs to be elaborated in more detail to enable a review. Also the spatial configuration (based on edge and interior trees, and height sectors of trees) of the orchard needs to be described in more detail.
7. The paper aims to more accurately reflect the outcomes of animal activity for society. It is not clear how these results can be applied by other orchards. Is the amount of birds feeding in the orchard representative for other orchards?
8. Application of the results by farmers also depends on the assumptions used by the author. How sensitive are the results for the almond market price, the (increasing) acreage of almond plantations, variability in weather (and climate), the labour costs, the trend in mechanical shaking of trees? Do the results vary over years?
9. The paper describes a partial analysis of ecosystem services of almond eating birds, e.g. other services these birds provide for society are not included. A more economically grounded approach will be necessary for a more complete analysis of these services to advice farmers or to inform the society on the value of nature.

I have read this submission. I believe that I have an appropriate level of expertise to confirm that it is of an acceptable scientific standard, however I have significant reservations, as outlined above.

# Response

**Published:** 2014-04-24

**Authors:** Gary Luck

**Affiliations:** Department of Wildlife Ecology and Management, Charles Sturt University, Australia

I thank Dr Reinhard for taking the time to review this paper, and for his constructive comments on the study. I have responded to each comment below and made changes to the paper where required to address relevant concerns.

 

1. I believe the current title of my paper is most appropriate given the context of the study. The work I present is not an examination of trading off costs and benefits from ecosystem services. The animal activity in question (eating almonds) is only an ecosystem service in one context. In another context it inflicts costs to growers through damage to almonds pre-harvest. It is very important that this distinction remains clear rather than referring to the activity of eating almonds as just an ecosystem service.
2. I set out the context of the study in the following paragraph:
* “The expansion of the almond industry raises substantial production and conservation management challenges. In Australia, almond crops attract a number of native bird species, especially parrots, which eat almonds during the growing season and reduce crop yield. However, the same bird species provide an ecosystem service to growers by eating residual nuts left on trees after the main crop has been harvested. These so-called ‘mummified nuts’ (mummy nuts) are susceptible to fungal infection, which may threaten future crop yields. Moreover, recent evidence shows that mummified nuts are used intensively by the carob moth (Ectomyelois ceratoniae) for food and breeding (http://australianalmonds.com.au/industry/conference_2012/proceedings). The carob moth is a pest of global significance, impacting the production of numerous crops worldwide including dates, figs, pistachio, citrus and pomegranate. It is a major emerging threat to the Australian almond industry, as it feeds on almond kernels rendering them unsuitable for human consumption. Mechanical or manual removal of mummy nuts post harvest is one approach to controlling moth outbreaks and fungal infections. However, birds are already providing this service to Australian growers – the question addressed in my study is whether the monetary value of this service outweighs the costs of bird damage to almonds, resulting in a positive net return from bird activity.” *
  I argue that the results of a net return analysis should be used to guide the level of investment required in animal management in a given system. For example, if the net return of bird activity is positive then it would be unnecessary for growers to invest money in controlling bird activity. I raise this issue and provide an example in the context of the pest control strategy of shooting to scare as follows:
*“In almond plantations, birds are currently considered pests and are subject to control strategies such as shooting to scare. In my study, shooters were employed for an average of 120 days per season over the growing seasons of 2009/10 and 2010/11. Based on an 8-hr day and minimum wage ($15.96 hr ^-1^), the cost of employing shooters is at least $15,322 season ^-1^. Over a 15,500 ha plantation (the entire plantation estate for my study) this equates to about $1 ha ^-1^, and appears to be a good investment if compared only to the cost of bird damage. For example, if shooting reduces bird damage by only 1% it would save growers $22.19 ha ^-1^. Yet, in the context of an overall positive net return from bird activity, the investment in shooting may be completely unnecessary.” *
3. Please see my response to Point #2 from Dr Nelson's review, and the new text I have added in regards to the problems associated with netting whole trees. It was not my aim to analyse differences in bird activity with or without shooting to scare birds. It is well recognised that shooting to scare has minimal impact on bird activity (see citations 20, 26 and 27) as they quickly become habituated to this practice. Attempting a controlled experiment with and without shooting would be extremely difficult in my study area given shooting is conducted on an *ad hoc* basis and the sound of a gunshot travels large distances (and hence would compromise any ‘treatment’ sites supposedly free of shooting activity).
4. Unfortunately, this detailed information is not recorded in a systematic way by growers and differs across different farms. Therefore, it was more appropriate to use the conservative estimate I included in my analysis, with the examination of how net returns vary under different cost and benefit scenarios (Figure 5 and related text).
5. I discuss this issue at some length in the second paragraph of the Discussion, but I have added the following new text to clarify the issue further:
*“Nevertheless, if the acceptable quantity of mummy nuts that can be retained per tree is substantially higher than two, then the cost of mummy nut removal by growers is reduced even further.”*
6. I have expanded on the description of the edge/interior comparison and its related statistical analysis as follows:
*“A total of 120 trees were included in the experiment with 60 netted trees (treatment) and 60 open trees (control). Thirty netted trees and 30 open trees were located at the edge of almond blocks (the exterior two rows) and 30 netted trees and 30 open trees were located in the interior of almond blocks (the centre point furthest from the edge – approximately 300 m). An edge/interior contrast was included because bird activity tends to be highest close to block edges. Control and treatment trees were assigned systematically to maximize interspersion, and I maintained a minimum distance of four trees between each control and treatment in the same edge or interior row to avoid adjacency effects and spatial autocorrelation in damage impacts (e.g. netted trees impacting outcomes on non-netted trees).”*
*“I determined if there was a block or row location (edge/interior) effect on nut loss attributed to birds or non-bird factors pre- and post-harvest using a generalized linear model with a binomial response. In this case, the binomial outcome was either nut damaged/lost vs. nut intact. That is, I compared nut outcome among almond blocks and edge and interior rows pre-harvest and post-harvest for open trees where the outcome could be either nut damaged/lost to bird activity or nut remained intact. I conducted the same analysis for netted trees pre- and post-harvest where nut outcome was affected by non-bird factors (e.g. wind). Row location was nested within block for these analyses and both were treated as random factors and p ≤ 0.05 was considered statistically significant.”*
7. Does the referee mean other almond orchards or other horticultural orchards? I don’t claim that the specific results I present are going to be transferable across a range of situations. That was never my intention. The aim of the paper is to present a case study of cost-benefit trade-offs from the activity of a given suite of animals. I do argue, however, that calculating the net return from animal activity in agro-ecosystems should be much more widely applied. Currently, research on ecosystem services mostly ignores the costs of animal activity, and research on animal pests largely ignores the benefits of animal activity. This has to change for a more holistic understanding of the outcomes of animal behaviour in different agricultural landscapes.
  I've added the following new text to the Discussion to make this clearer (second to last paragraph):
*“The specific results of the case study I present here may not be transferable across different contexts. Nevertheless, the need to calculate a net return from animal activity is broadly relevant because there are few situations in nature whereby the activity of animals in a given location is entirely beneficial or entirely detrimental – especially when considering a broad range of taxa.”*
8. See my response to Point #4 from Dr Nelson in regards to varying the market price of almonds including new text and new trend lines in Figure 5. Figure 5 shows trends in net returns given different labour costs, bird damage rates, time spent hand poling, different mechanical shaker rates etc.
9. This is a great point and one I tried to highlight with the final paragraph of my Discussion as follows:
*​“I've focused on a small suite of species and a single activity to clearly illustrate the net return from animal activity. Analyses could be extended to encompass more species and more activities, dependent on available ecological knowledge. For example, I have recorded 35 insectivorous bird species using almond orchards in my study area that may contribute to controlling carob moth. Almond flowers require cross pollination to set seed, and in north-west Victoria, over 100,000 European honeybee (Apis mellifera) hives are trucked into almond plantations each year, costing growers more than $7 million annually. Yet, almost nothing is known of the potential contribution that native pollinators could make to almond pollination in this region. A more complete analysis could consider the costs and benefits, and ultimately net return, of the activities of a range of species.”*

# Peer review: Referee response for version 1

**DOI:** 10.5256/f1000research.2837.r2406

**Published:** 2013-12-09

**Authors:** Erik Nelson

**Affiliations:** 1 Department of Economics, Bowdoin College, Brunswick, ME, USA

The general framework of the paper seems to be appropriate: 1- Calculate the monetary benefit of birds feeding in almond patches (“free” predation of mummified almonds by birds in lieu of costly human removal); 2- add up the cost of these birds in almond patches (predation of harvest-able almonds by birds); 3- subtract the value of lost harvest-able almonds from the cost of human and/or machine work avoided. If the resulting value is greater than 0 then birds on Australian almond patches are, in net, beneficial to society and not a pest in almond systems (assuming the investigated almond patch is representative of other Australian almond farms).

Luck’s main conclusion is that birds are more likely to be a benefit to society and not a pest in aggregate IF 1) they cause the more expensive hand-poling (versus mechanical shaking) of trees to be avoided and 2) we focus on the birds’ impact on the edges of almond plots (in Luck’s experiment birds did not seem to penetrate to the interior of the almond patch).

My observations and concerns with the methodology:

1. What about the combination of bird and human control of mummified almonds? What is the optimal level of human input to mummified almond control given the free input provided by birds?  For example, could farmers use GPS technology to determine where to best compliment beneficial bird activity? Is there a way to quickly and relatively cheaply determine where bird activity needs to be supported? Up to this point too much of the “ecosystem service on farms” literature has presented animal input as an either/or option (e.g., wild pollinators versus rented hives on animal pollination-limited crops). We need to start looking more and more at the OPTIMAL combination of human and natural input (e.g., farmers making this type of calculation when they decide how much nitrogen fertilizer to add to a field given a map of pre-existing nitrogen levels). How would we go about determining the optimal management of almond farms where birds are explicitly considered to be part of the system? How would the planting of decoy crops for birds on the periphery of the almond farm contribute to optimal management of farms?
2. In the experiment Luck only nets branches and not whole trees. An experiment with whole trees netted would seem to be more appropriate. Luck’s explanation for not netting whole trees needs to be better defended in the text, and the potential issues with branch netting versus whole tree netting on experimental results need to be more fully considered.
3. Luck relies a lot on linear extrapolation. For example, he found *“mean nut loss from netted trees was 5.9%...so, if for example, 50 nuts were completely lost from an almond tree, I considered that three nuts (5.9%) were lost due to non-bird factors…” *.  First, he must mean that 5.9% were lost from the netted branches, not trees, as loss from branches was controlled, not whole trees. Second, is such a simple linear extrapolation from branch loss to tree loss appropriate? Luck thinks so, I am not so sure. I would like to see this issue investigated further.
4. The market price of almonds can vary wildly year to year, just like any crop. Luck only uses market prices from one year to generate his conclusions. He needs to consider a greater range of prices in a sensitivity analysis.
5. Luck needs to give us an idea of the relative use of manual hand-poling versus mechanical shaking of trees on almond farms now and in the future. Do most farmers mechanically shake trees? What is the trend? Is mechanical shaking becoming cheaper and more widespread over time? Information on this trend would seem to affect the direction of study into the impact of birds in the almond system. If mechanical shaking is becoming the norm than birds are likely to be, in net, a pest in the future and the most animal-friendly management of this pest should become the focus of research. Further, what are the future projections for almond prices? This would also affect conclusions on promising future research directions.

I have read this submission. I believe that I have an appropriate level of expertise to confirm that it is of an acceptable scientific standard.

# Response

**Published:** 2014-04-24

**Authors:** Gary Luck

**Affiliations:** Department of Wildlife Ecology and Management, Charles Sturt University, Australia

I thank Dr Nelson for taking the time to review this paper, and for his insightful comments on the study. I have responded to each comment below and made changes to the paper where required to address relevant concerns.

1. This is an excellent point – and, as the referee notes, a criticism that can be leveled at numerous studies of ecosystem services. I actually believe that I dealt with the issue of how both bird and human control of mummy nuts will likely be required when I highlighted the fact that birds are only removing a proportion of mummy nuts prior to the next almond flowering. Please see the last two paragraphs of the Results where I demonstrate that birds were removing only a certain percentage of mummy nuts and how this translated into reduced costs to growers. Implicit in this is that if growers want 100% of mummy nuts removed, they are going to have to employ mechanical or manual methods to supplement bird activity. I also raise this issue at various points in the Discussion, but maybe I wasn’t explicit enough (see new text below).
  I’m not sure I am comfortable in making pronouncements about a definitive ‘optimal’ combination of human and natural input. It is important to recognise that ecosystem service input will vary from one year to the next owing to changing environmental conditions. For example, bird numbers and activity in almond orchards can vary significantly with rainfall ( Luck *et al.,* 2013) Moreover, the cost of bird damage will vary with fluctuations in the market price of almonds, and the value of the ecosystem service will differ with fluctuations in replacement costs (e.g. change in salary rates).
  Hence, any optimal combination will vary from one year to the next. I argue that it is less about identifying an optimal combination (if it indeed exists) and more about understanding how cost-benefit trade-offs will change given different environmental conditions (affecting ecosystem-service delivery) and fluctuating monetary values (of both costs and benefits). Key to this is recognising what factors need to be measured or monitored each year so that growers can make informed decisions about orchard management that will minimise costs, maximise benefits, and not undermine current and future potential for ecosystem-service delivery.
  To highlight this issue properly, I have added the following text to the Discussion:
*"The fact that birds did not remove all mummy nuts prior to the next almond flowering highlights the important point that ecosystem-service provision in this case does not replace completely the need for human input to achieve acceptable outcomes. A combination of human action and ecosystem-service provision is required, and I suggest this is likely to be the case in many different systems. It is important to determine, therefore, how the proportional contribution required from human action or other organisms changes with different environmental, social and economic conditions. For example, in my study, because bird activity was highest at crop edges, the proportional contribution by farm managers to mummy nut control may be minor in this location, but increase further from the edge.” *
2. I have added the following new text to the methods section (3 ^rd^ paragraph under ‘Costs’).
*“Netting whole trees was problematic and could have compromised the integrity of the experiment in several ways. Netting the entire tree would have disrupted regular orchard maintenance such as selective pruning and the spraying of herbicides to control weed growth. Hence, netted trees would have differed from non-netted trees in more than just the presence of a net. Second, strong wind is locally common and can be particularly problematic at orchard edges, which have little protection from the elements. Given the structure of almond trees, and the closeness of plantings, there was a real threat of nets ripping during strong winds if placed over the entire tree. Third, the larger the area netted, the more likely that birds will find gaps in netting. It is extremely difficult to ensure that birds are completely excluded from an entire tree even when careful attention is paid to closing all visible gaps in the net. Owing to these concerns, I decided that netting a single branch was a more acceptable method to employ. I do not believe that this approach undermines the general conclusions of the study.”*
3. To avoid confusion, I have re-worded the relevant sentence as follows:
*“Mean nut loss from netted trees (i.e. netted branches) was 5.9% (± SEM 0.82%, n = 60), so if, for example, 50 nuts were completely lost from an open tree [branch], I considered that three nuts (5.9%) were lost due to non-bird factors and the remainder due to birds.”*
  It is important to note that in this particular sentence I am not extrapolating values from branches to whole trees, but using nut loss from netted branches caused by non-bird factors (e.g. wind, tree health) to determine what percentage of nuts from open branches were likely to also have been lost owing to non-bird factors. Later in the paper, I do extrapolate yield losses per branch to whole trees based on values obtained from a related study ( Luck et al., 2013) that measured the actual number of nuts across 200 trees. While this extrapolation is linear, it is the best estimate available given current knowledge of bird activity and damage in almond orchards.
4. This is a valid point, but it is a criticism that can be leveled at every study that attempts to value costs or benefits of animal activity in crops in monetary terms. In this comment though, the referee only acknowledges one side of the equation in relation to the current study. Not only will market prices for almonds fluctuate, but so will the costs associated with replacing the ecosystem service such as the running costs of the mechanical shaker or the salary costs of orchard workers. Hence, any sensitivity analysis, should it be required, would need to consider both the costs of bird activity as market values of almonds change, and the ecosystem-service value of their activity with changes in the replacement costs of the service. This may or may not yield a substantially different net return. I had attempted to present this cost-benefit trade-off given differing costs of damage and value of the ecosystem service in Figure 5. This figure is, in essence, a sensitivity analysis of the cost-benefit and net return relationship given variation in monetary amounts, though I acknowledge that altering the market price of almonds was not part of the initial analysis.
  Therefore, to address this concern, I have added two new trend lines to Figure 5 which show how cost-benefit trade-offs vary under different market prices for almonds based on the expected 2013 price and a hypothetical 2014 price assuming the price increase from 2013-14 is equivalent to the increase from 2012-13. 
  The purpose of my study was to argue for greater attention to be paid to cost-benefit trade-offs associated with animal activity in agriculture and to present an example of how these cost-benefit trade-offs could be examined. I would assume that if researchers in the future adopt my methods they will substitute the most relevant market prices and replacement costs etc. to calculate the most appropriate net return for their given situation.
  To address this issue further I have added the following text to the Discussion:
*“It is also important to note that the sign and the magnitude of the net return of animal activity will vary with fluctuations in the market prices for crops and the costs of replacing the ecosystem service (e.g. the running costs of machinery and the salary costs of orchard workers). An analysis of how cost-benefit trade-offs change with varying monetary values is provided in Figure 5, including trend lines based on different market prices of almonds. Researchers that adopt the approach of calculating a net return from animal activity should use the most recent and relevant monetary values for their specific situation.”*
5. Attempting to predict the future is always fraught with danger, so I do not believe it is appropriate to speculate on how almond prices or farm management will vary in coming years. The Australian almond industry is very reliant on the availability of water for irrigation, and in Australia, rainfall from year to year can be highly unpredictable and this will impact on farm management. Also, different farms are managed in different ways and it is not appropriate to generalise across numerous farms. Moreover, the threat of the carob moth has only recently been acknowledged and its presence in orchards may greatly alter orchard management into the future.
